# Autologous Muscle-Derived Nerve Wrap for Prevention of Symptomatic Microneuromas in Primary Nerve Repair

**DOI:** 10.7759/cureus.22513

**Published:** 2022-02-23

**Authors:** William J Bruce, Amanda L Brown, Michael R Romanelli, Brian A Mailey

**Affiliations:** 1 Department of Surgery, Institute for Plastic Surgery, Southern Illinois University School of Medicine, Springfield, USA

**Keywords:** microneuroma, muscle wrap, nerve wrap, regenerative peripheral nerve interface, nerve repair, peripheral nerve, neuroma

## Abstract

Regeneration of peripheral nerves after repair is incomplete. Painful microneuromas may form at the site of an appropriately performed primary microsurgical nerve repair leading to a persistent Tinel’s sign and hypersensitivity in that location. Here, we describe an autologous option using a free muscle-derived nerve wrap with the intent to capture axonal escape at the site of primary nerve coaptation. We demonstrate this technique on a patient undergoing primary nerve repair of a laceration to the superficial branch of the radial nerve using extensor digitorum communis muscle as a donor graft. This has become our preferred technique over commercially available nerve wraps as the muscle wrap is autologous, not limited by cost, and has the potential to limit microneuroma formation at the coaptation site.

## Introduction

Peripheral nerve injuries are common and diagnosed between 1% and 5% of patients treated for traumatic injuries [1]. The gold-standard reconstruction after transection of a peripheral nerve is tension-free primary repair. Axonal regeneration after primary coaptation is consistently incomplete. With an ideal repair, each coaptation site experiences a 30-50% reduction in axon count distal to the neurorrhaphy [2]. This decrease in axon density is thought to result from perineural scarring, decreased Schwann cell activity, and “axon escape”- better described as misdirected axon sprouting - all of which impede distal axon growth and signal transduction and can lead to the formation of painful neuromas [3]. Up to 45% of patients will develop some degree of chronic pain, scar hypersensitivity, or cold intolerance directly related to these changes, both in the presence or absence of a clinical neuroma [4]. These symptoms often continue to occur at the proximal repair site despite regeneration of the distal nerve. In addition to being painful, this can present with duplication of the Tinel’s sign at the repair site which makes monitoring clinical progression of nerve healing difficult. We hypothesize that this is due to the formation of microneuromas as a direct result of the axon escape that occurs at each coaptation.

Commercial nerve wraps can be used as an adjunct to primary repair and are intended to decrease perineural scarring and improve distal axon regeneration resulting in better distal functional recovery [3,5]. However, these wraps are not demonstrated to prevent the formation of neuropathic pain at the coaptation site.

Modern treatments for prevention of clinically apparent neuroma formation redirect a proximal nerve after transection into a segment of denervated muscle or dermis to prevent further aberrant growth [6-8]. Creation of a regenerative peripheral nerve interface (RPNI) or dermatosensory peripheral nerve interface (DSPNI) is a versatile and effective technique for the treatment and prevention of neuromas and associated pain using readily available donor muscle or dermis. Original descriptions of RPNI free muscle grafts were 1.0cm x 1.0cm x 0.3cm in the hand and 3.0cm x 1.5cm x 1.0cm in the major lower extremity nerves [6,9]. These sizes remain viable as a free graft and maintain the potential for reinnervation. Additionally, studies of the muscle cuff (mc)-RPNI placed around an intact nerve for signal amplification in prosthetic control-show promise in their ability to reinnervate and remain viable while avoiding nerve constriction, muscle graft fibrosis, scar formation, neuroma formation, or distal signal attenuation [10].

Based on the well-studied benefits of RPNI combined with the need for an autologous nerve wrap option came the rationale for using free muscle-derived grafts at the time of nerve repair. Here, we present the biologic autologous muscle-derived nerve (BAM)-wrap intended for use at the time of nerve repair when donor muscle is available within the surgical field. This technique can stabilize and prevent tension at the repair and may limit painful and hypersensitive microneuromas by drawing on the effectiveness of RPNIs for the prevention of neuroma formation.

## Technical report

Indications

The BAM-wrap can be employed both in acute repair of a traumatic nerve injury and in allo- or autograft-based reconstruction whenever a donor muscle is available in the surgical field. The clinical objective of this procedure is to capture misdirected axons at each coaptation site thereby preventing microneuroma formation and neuropathic hypersensitivity. Secondarily, this wrap directly prevents tension at the repair site and may behave in a similar fashion to commercially available nerve wraps by inhibiting perineural scarring and optimizing the environment for axonal regeneration [11].

Surgical technique

**Figure 1:**
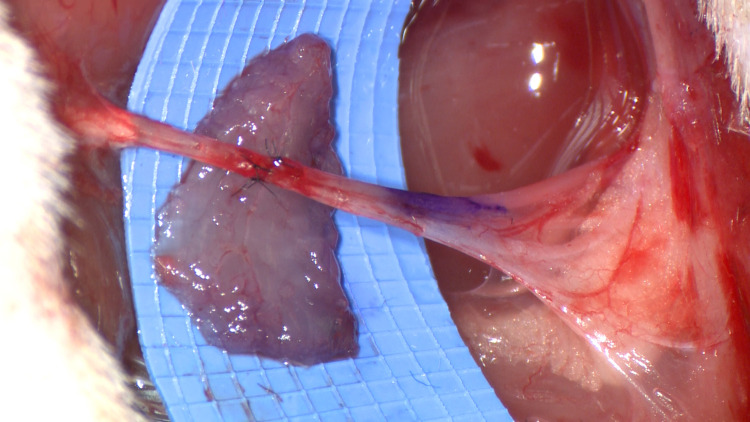
Free muscle graft prior to the wrap shown in rat sciatic nerve model.

The affected nerve is trimmed to healthy edges and repaired in a tension-free epineural fashion. A free muscle graft is then harvested from a healthy donor muscle within the surgical field (Figure 1). We have found 1cm x 1cm x 0.3cm to be sufficient size for use in the hand and fingers, with slightly larger cuffs (1.5-2cm) used to allow circumferential wrapping around major upper or lower extremity nerves. In a surgical site where there is no readily available donor muscle, such as an isolated digital nerve laceration, a similar-sized piece of de-epithelialized dermis can be used.

**Figure 2:**
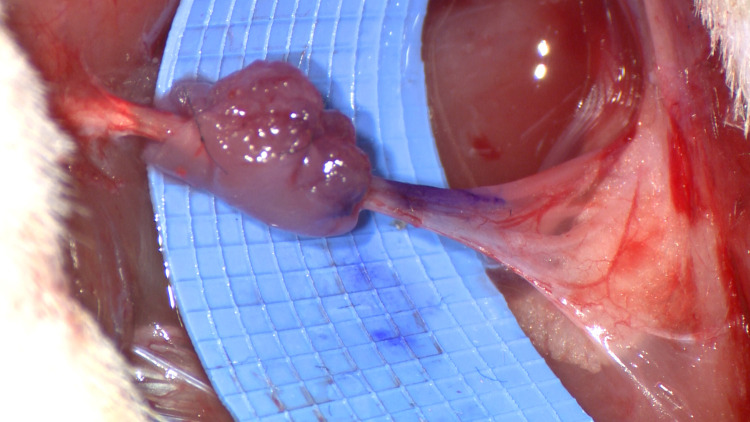
Muscle wrap applied to rat sciatic nerve model after primary repair.

The graft is sutured into a cuff around the coaptation site with a 7-0 nylon suture. Three sutures are sufficient to maximize contact between the graft and the repaired nerve. The proximal and distal sutures are passed through epineurium to maintain the position of the graft and prevent tension on the coaptation (Figure 2, Video 1). Fibrin glue can be applied to the construct to further stabilize the position of the graft. We routinely utilize previously described adjuncts to nerve coaptation such as intraoperative nerve stimulation and induced proximal crush injury to optimize distal regeneration after repair [12].

**Video 1:** Nerve coaptation with biologic autologous muscle wrap. The muscle-cuff nerve wrap technique is demonstrated on microsurgery teaching animal model (rat sciatic nerve transection) after primary microsurgical repair. A local muscle is harvested, trimmed to the appropriate size, and fashioned into a cuff around the coaptation site.

## Discussion

**Figure 3:**
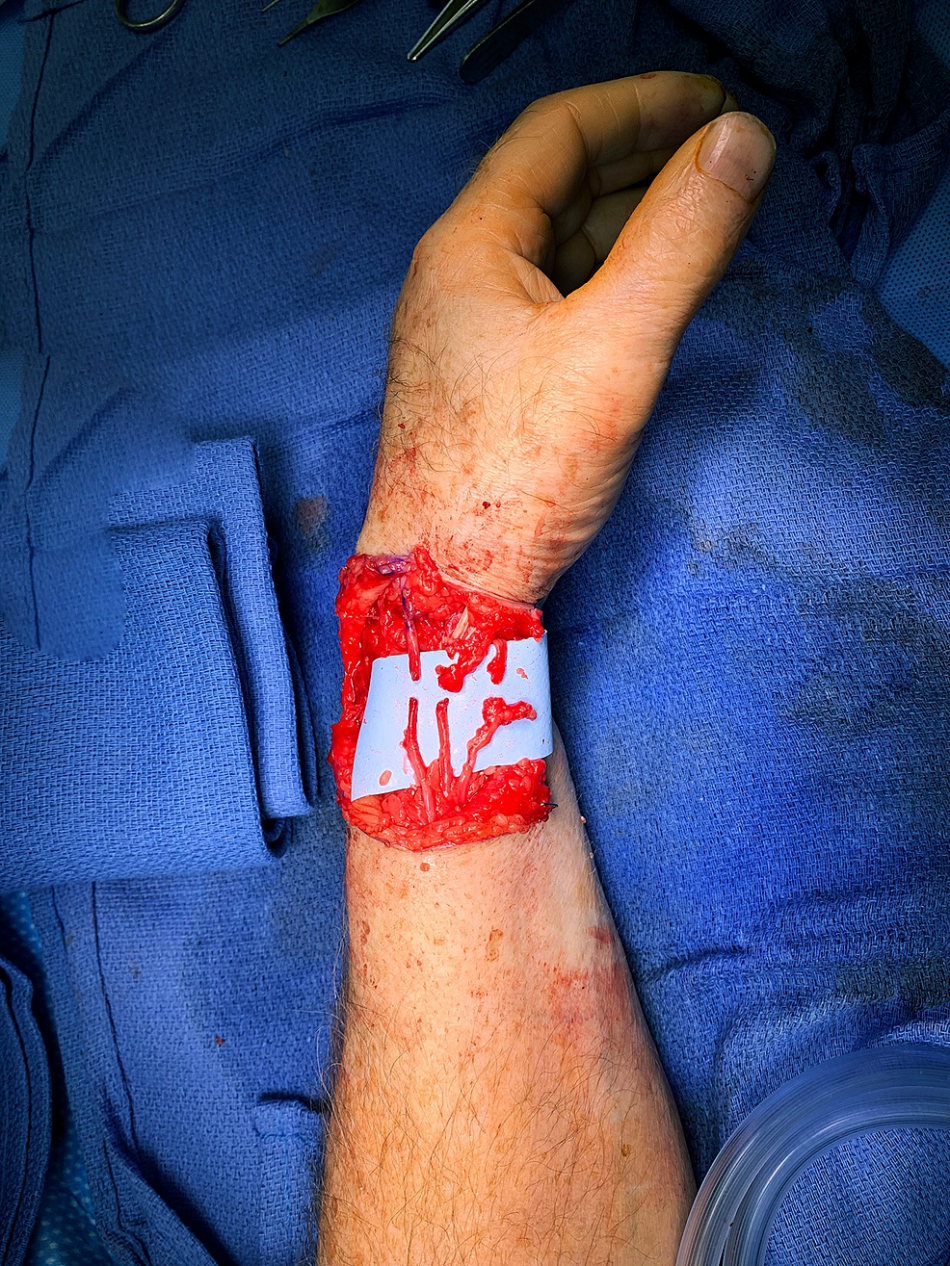
Superficial radial nerve injury at wrist. A 57-year-old male sustained a sharp laceration to the radial wrist involving three branches of the superficial radial nerve (SBRN). The superficial nature of this nerve predisposes it to symptomatic microneuroma formation.

**Figure 4:**
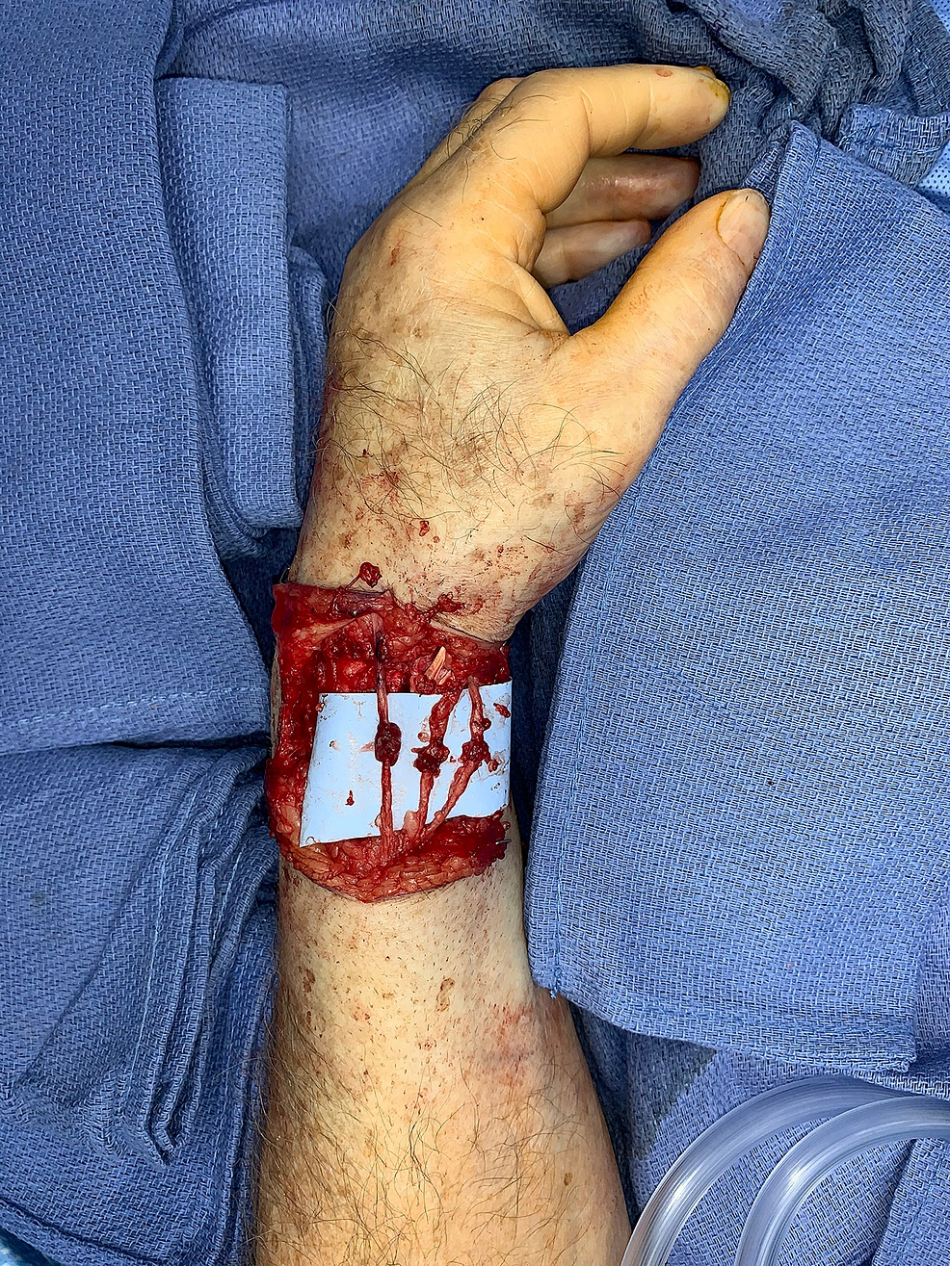
Repair of superficial branch of radial nerve augmented with BAM-wrap. The patient was taken to the operating room and the lacerated nerve branches were repaired primarily. A BAM-wrap was fashioned from the extensor-digitorum communis (EDC) muscle belly within the surgical field and applied to each of the three superficial radial nerve (SBRN) coaptation sites.

The BAM-wrap has become our preferred supplement to nerve repairs with the goals of preventing tension or stress on the repair, maximizing the return of function, and limiting the development of postoperative neuropathic pain. We have used this technique in upper and lower extremities in the setting of acute primary repairs after trauma, after resection of neuromas-in-continuity, and combined with targeted muscle reinnervation (TMR) after resection of symptomatic end-neuromas (Figures 3, 4). We intend to continue the long-term analysis of these patient outcomes. We hypothesize that findings of the mc-RPNI will be reproduced, namely reinnervation and viability of the muscle graft, no reduction in distal axon counts or motor function, and lack of subsequent perineural scarring or constriction [10].

## Conclusions

The known benefits of forming a regenerative peripheral nerve interface to prevent neuroma pain and augmenting a coaptation with a nerve wrap together make this technique a straightforward, cost-effective, and readily available adjunct to primary nerve repair. The regular use of a muscle wrap may augment primary nerve repair by directly preventing tension on the repair and limiting post-operative pain through targeted redirection of aberrant nerve growth.
