# Correction: Autologous Muscle-Derived Nerve Wrap for Prevention of Symptomatic Microneuromas in Primary Nerve Repair

**DOI:** 10.7759/cureus.c98

**Published:** 2023-01-25

**Authors:** William J Bruce, Amanda L Brown, Michael R Romanelli, Brian A Mailey

**Affiliations:** 1 Department of Surgery, Institute for Plastic Surgery, Southern Illinois University School of Medicine, Springfield, USA

This article has been corrected at the request of the authors to remove the following sentence from the discussion section:

“Additionally, a parallel study of the direct physiologic effects of this technique on an animal model - and comparison to commercial nerve-wrap products - is ongoing in our laboratory.”

Such a study is not ongoing in the lab and has not been completed.

The authors deeply regret that these errors were not identified and addressed prior to publication.

